# Detection of early alterations in radiologically normal-appearing brain regions before recurrence of high-grade glioma using multiparameter mapping and diffusion tensor imaging

**DOI:** 10.1093/noajnl/vdag173

**Published:** 2026-07-02

**Authors:** Philipp Raffler, Severin Schramm, Cornelius Berberich, Daniele Calabro, Ronja Berg, Kirsten Jung, Julian Ziegenfeuter, Tim Herrmann, Claire Delbridge, Stephanie C Combs, Bernhard Meyer, Christine Preibisch, Benedikt Wiestler, Marie-Christin Metz

**Affiliations:** Department of Neuroradiology, TUM School of Medicine and Health, TUM Klinikum Rechts der Isar, Technical University of Munich, Munich, Germany; Department of Neuroradiology, TUM School of Medicine and Health, TUM Klinikum Rechts der Isar, Technical University of Munich, Munich, Germany; Department of Neuroradiology, TUM School of Medicine and Health, TUM Klinikum Rechts der Isar, Technical University of Munich, Munich, Germany; Department of Neuroradiology, TUM School of Medicine and Health, TUM Klinikum Rechts der Isar, Technical University of Munich, Munich, Germany; Department of Neuroradiology, TUM School of Medicine and Health, TUM Klinikum Rechts der Isar, Technical University of Munich, Munich, Germany; Department of Neuroradiology, TUM School of Medicine and Health, TUM Klinikum Rechts der Isar, Technical University of Munich, Munich, Germany; Department of Neuroradiology, TUM School of Medicine and Health, TUM Klinikum Rechts der Isar, Technical University of Munich, Munich, Germany; Department of Neuroradiology, TUM School of Medicine and Health, TUM Klinikum Rechts der Isar, Technical University of Munich, Munich, Germany; Department of Radiation Oncology, TUM School of Medicine and Health, TUM Klinikum Rechts der Isar, Technical University of Munich, Munich, Germany; Department of Neuroradiology, TUM School of Medicine and Health, TUM Klinikum Rechts der Isar, Technical University of Munich, Munich, Germany; Department of Neuroradiology, TUM School of Medicine and Health, TUM Klinikum Rechts der Isar, Technical University of Munich, Munich, Germany; Department of Neuroradiology, TUM School of Medicine and Health, TUM Klinikum Rechts der Isar, Technical University of Munich, Munich, Germany; TranslaTUM, TUM School of Medicine and Health, TUM Klinikum Rechts der Isar, Technical University of Munich, Munich, Germany; AI for Image-Guided Diagnosis and Therapy, School of Medicine and Health, Technical University of Munich, Munich, Germany; Department of Neuroradiology, TUM School of Medicine and Health, TUM Klinikum Rechts der Isar, Technical University of Munich, Munich, Germany

**Keywords:** DTI-derived free water, high-grade glioma, multiparameter mapping, quantitative MRI, recurrence

## Abstract

**Background:**

Quantitative MRI (qMRI) might detect subtle changes in recurrent high-grade glioma earlier than conventional imaging. The purpose of this study was to investigate whether WHO grade 4 glioma patients demonstrate alterations in multiparameter mapping (MPM) and diffusion tensor imaging (DTI)-derived measures within radiologically normal-appearing brain regions that subsequently exhibit tumor recurrence in follow-up scans.

**Methods:**

For 16 WHO grade 4 glioma patients with confirmed recurrence at follow-up, qMRI parametric maps [proton density (PD), longitudinal relaxation rate (R1), transverse relaxation rate (R2*)] were generated using an MPM protocol. Additionally, diffusion tensor imaging (DTI)-derived free water (FW) and FW-corrected tissue fractional anisotropy (FAt) were evaluated. We mapped recurrent tumor areas onto baseline scans to identify regions that subsequently progressed to contrast-enhancing tumor (CET) or FLAIR-hyperintense areas.

**Results:**

Normal-appearing gray matter (NAGM) that progressed into FLAIR-hyperintense areas in follow-up scans showed significantly lower median PD values, while median FW was significantly elevated across all subregions compared to their reference regions. This potentially reflects an early redistribution of water from bound tissue compartments into the extracellular space, most evident in gray matter. R1 demonstrated significantly lower median values in NAGM progressing to tumor compared to stable NAGM. Variance was significantly increased for PD, R2*, and FW in multiple subregions, consistent with heterogeneous tumor infiltration.

**Conclusions:**

MPM and DTI-derived metrics reveal subtle alterations in radiologically normal-appearing brain tissue months before recurrence becomes visible on conventional imaging. This could support earlier identification of subclinical progression and allow for personalized surveillance strategies.

Key Points

- In high-grade glioma, MPM and DTI-derived free water reveal changes in normal-appearing brain tissue months before recurrence.
- Future recurrence shows lower proton density but higher free-water fraction, indicating a potential early compartmental water shift.

Importance of the Study

Early detection of high-grade glioma recurrence remains a major clinical challenge, as conventional MRI relies on contrast enhancement and FLAIR abnormalities that emerge only after substantial microstructural damage has occurred. This study demonstrates that quantitative relaxometry (PD, R1, R2*) and DTI-derived metrics reveal consistent, spatially localized alterations in radiologically normal‑appearing brain tissue months before recurrence becomes apparent on conventional imaging. The observed combination of reduced proton density, subtle R1 and R2* changes, and increased free‑water fraction supports a biologically plausible model of compartmental water redistribution driven by early tumor cell infiltration and demyelination. These quantitative MRI measures may therefore help to identify subclinical progression and enable more personalized surveillance strategies and earlier treatment decisions.

High-grade gliomas, particularly WHO grade 4 glioblastomas, are among the most frequent and aggressive primary malignant brain tumors in adults, with a 5-year survival rate of 9.8% for glioblastoma patients despite maximal therapeutic intervention.^1^^,^^2^ Recurrence is common, with a median progression-free survival of 6.9 months.^3^ Conventional response assessment criteria, such as RANO (Response Assessment in Neuro-Oncology) 2.0, rely on contrast enhancement.^4^ Recurrence, however, may occur before these changes are considered “measurable disease” on standard MRI sequences.^5^

Conventional MRI techniques have critical limitations for predicting early recurrence. Signal intensities are scanner-dependent and arbitrary, thereby precluding reliable quantitative comparisons across platforms or time points.^6^ Quantitative MRI relaxometry, with values such as the longitudinal relaxation rate (R1), effective transverse relaxation rate (R2*), and proton density (PD), overcomes these constraints by providing absolute measurements of tissue properties that are independent of scanner parameters and intrinsically reproducible.^7^^,^^8^ R1 and R2* are the inverse of the longitudinal relaxation time (T1; R1 = 1/T1), and the effec­tive transverse relaxation time (T2*; R2* = 1/T2*), respectively.

Diffuse glioma infiltration extends beyond macroscopically visible margins, with microscopic tumor cells infiltrating normal-appearing brain regions.^9^ Emerging evidence suggests that areas of future recurrence exhibit subtle microstructural and vascular changes, as detected by advanced imaging techniques such as diffusion-tensor imaging (DTI)^10^ or multiparametric analyses including dynamic susceptibility contrast (DSC)-perfusion.^11^

These changes might also be detectable via quantitative relaxometry. Subtle blood-brain-barrier (BBB) disruptions were found in subtraction maps of quantitative R1 maps before and after gadolinium application.^12^^,^^13^ Progressive disease in glioblastoma might also manifest through prolonged T1^14^ or decreased T2 relaxation times.^14^^,^^15^

Early recurrence detection carries substantial clinical implications. The clinical evidence for reoperation in recurrent glioblastoma shows a survival benefit primarily when recurrence occurs late (interval >6-9 months). In contrast, early recurrences (<6 months) reflecting aggressive tumor biology often lack a significant survival advantage from surgery alone.^16^ Preservation of a good functional status at the time of reoperation remains a strong predictor of overall survival. This underscores the clinical imperative to detect recurrence before symptom onset as it may extend the window for multimodal interventions—such as re-resection, re-irradiation, or trial enrollment—prior to loss of treatment eligibility. In parallel, timely integration of palliative care supports quality of life and anticipatory care planning.^17^

The purpose of this study was to investigate whether patients with WHO grade 4 glioma demonstrate quantifiable alterations in quantitative MR parameters within radiologically normal-appearing brain regions that subsequently progress to recurrence, as detected by follow-up scans.

## Methods

### Study Population

A database of 126 glioma patients (49 female) who received quantitative MRI at our institution was established from September 11, 2019, to August 4, 2025, comprising a total of 190 imaging timepoints. The cohort included both newly diagnosed patients at initial presentation and postoperative follow-up cases.

### Data Extraction and Processing

The imaging protocol can be found in Supplementary Files (S1). MRI data of eligible patients were extracted from the institutional PACS system. The quantitative MPM data were processed using the hMRI Matlab Toolbox^18^ to create the PD-, R1-, and R2*-maps as described previously.^7^

Diffusion-weighted images were preprocessed using the *PreQual* pipeline (MASI Lab),^19^ which integrates tools from FSL, ANTs, and FreeSurfer. In brief, diffusion data were denoised and corrected for eddy current-induced distortions, subject motion, and slice-to-volume motion using FSL *eddy* with outlier detection and replacement. Diffusion gradient directions were rotated accordingly. A synthetic B0 image was generated and combined with TOPUP to estimate susceptibility-related deformations, which were used to improve diffusion-to-structural image registration.

The free-water (FW) analysis was performed using a customized MATLAB script.^20^ Briefly, a bi-tensor model was fitted voxel-wise to separate the diffusion signal into a FW component and a tissue compartment. This resulted in a FW-corrected fractional anisotropy map (FAt) and a free-water map (FW), representing the fractional volume of free water per voxel ranging from 0 to 1.

To quantify free-water content in the investigated regions, subject-specific structural masks were transformed into native diffusion space using ANTs-derived registration. Nearest-neighbor interpolation was applied to preserve ROI integrity. Free-water diffusion metrics were subsequently extracted within each ROI.

MRI data from baseline (*t*_0_) and recurrence (*t*_1_) timepoints were co-registered using FSL’s linear registration tool with six degrees of freedom, applying mutual information as the cost function and trilinear interpolation for spatial resampling. All images at *t*_1_ were automatically segmented into necrosis, contrast-enhancing tumor (CET) on T1w post-contrast (T1c), and FLAIR-hyperintense areas using the BraTS Toolkit,^21^ and manually refined in ITK-SNAP.^22^ In the *t*_0_-scans, normal-appearing white matter (NAWM), normal-appearing gray matter (NAGM), and cerebrospinal fluid (CSF) were automatically segmented using CAT12 using the native T1-weighted sequence as input.^23^ These ­segmentations encompass the entirety of NAGM and NAWM across both hemispheres. As this study focuses on normal-appearing brain tissue, preexisting FLAIR-hyperintensities, contrast-enhancing regions, scar tissue, and regions with extensive white matter lesions were manually excluded. The two segmentations were merged and coregistered to the *t*_0_ space using FSL to delineate the following subregions:

- NAGM/Necrosis: NAGM in *t*_0_, necrosis in *t*_1,_
- NAWM/Necrosis: NAWM in *t*_0_, necrosis in *t*_1,_
- NAGM/CET: NAGM in *t*_0_, CET in *t*_1_,
- NAWM/CET: NAWM in *t*_0_, CET in *t*_1_,
- NAGM/FLAIR: NAGM in *t*_0_, T2/FLAIR hyperintense areas in *t*_1_,
- NAWM/FLAIR: NAWM in *t*_0_, T2/FLAIR hyperintense areas in *t*_1_.

**Figure 1.**
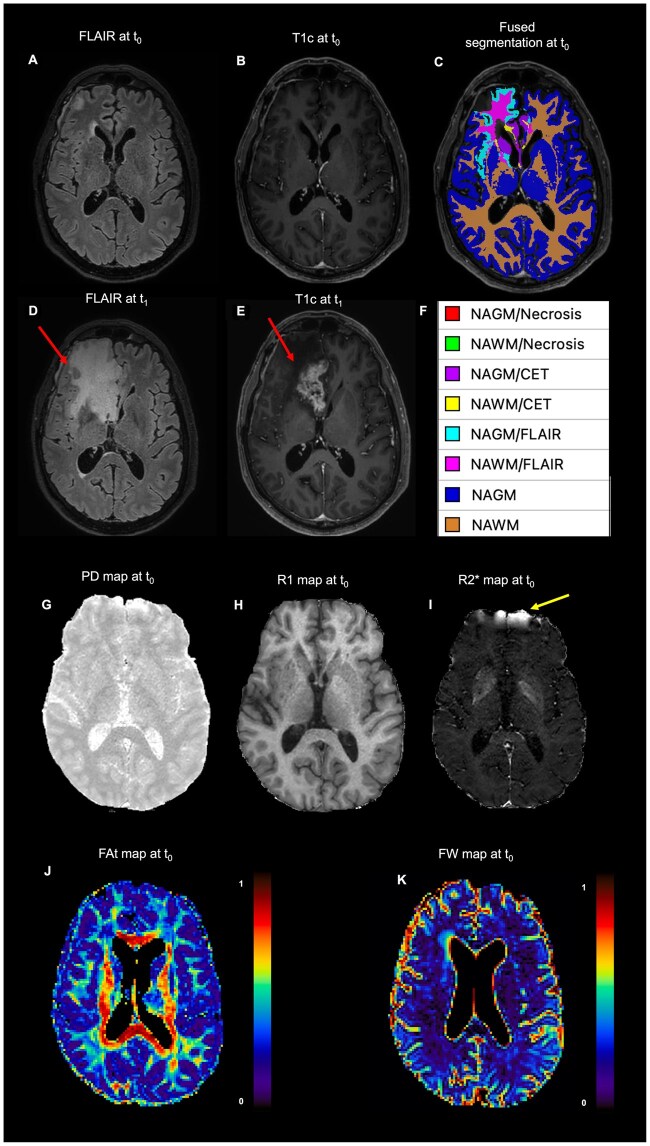
A 59-year-old male patient with glioblastoma WHO CNS grade 4: Routine scans for response assessment (*t*_0_) with no signs of tumor recurrence in axial FLAIRw sequence (A) and T1w post-contrast (T1c, B). Stable disease according to RANO 2.0. (D + E) The same patient 3 months later (t1). Extensive tumor recurrence with new FLAIR-hyperintensities, CET, and necrosis in the right frontal lobe (see arrows in D and E). Progressive disease according to RANO 2.0. (C) Fused segmentation of subregions shows normal-appearing tissue at *t*_0_ that will progress to tumor regions at (*t*_1_). Note that preexisting FLAIR-hyperintensities in the right frontal lobe and adjacent to the right lateral ventricle were manually excluded. (F) shows the different subregions. The violet label, for instance, depicts NAGM in the routine scan, which became CET in the follow-up scan. Quantitative maps at *t*_0_:(G) PD map, (H) R1 map, (I) R2* map. Note the susceptibility-artifacts close to the frontal sinuses in the R2* map ( arrow in I). DTI-derived metrics at t0: FAt map (J), FW map (K).

The fused segmentation underwent manual quality assurance with modifications applied as necessary and was used for statistical analyses of the quantitative maps in the baseline scans. Figure 1 shows the evaluated parameter maps of one exemplary patient. For R2*, additional manual refinements of the fused segmentations were performed, and regions that were affected by severe susceptibility-induced artifacts at air-bone-tissue interfaces were excluded from the analysis because of unreliable R2* estimation. These artifacts (mostly/usually) occurred adjacent to the skull base, particularly at frontal sinuses and mastoid air cells.

DSC perfusion was acquired as part of the routine clinical protocol but was not included in the present analysis, which was specifically focused on the less well-studied quantitative relaxometry and DTI-derived metrics.

### Statistical Analysis

Statistical analyses were performed using MATLAB version R2024b (The MathWorks Inc., Natick, Massachusetts, USA) with the SPM add-on for the extraction of descriptive statistics of the determined subregions [median, 5th percentile (P5) and 95th percentile (P95)]. Nonnormal distribution of PD, R1, R2*, FW, and FAt values was confirmed by visual analysis of histograms and Q-Q plots. Consequently, nonparametric Wilcoxon signed-rank tests were employed to compare quantitative imaging parameters across subregions. Additionally, the Fligner-Killeen test was used to assess variance heterogeneity, which is robust against non-normally distributed data. Multiple comparison correction was conducted using false discovery rate (FDR) adjustment. To identify potential trends below statistical significance thresholds, we calculated the rank-based effect size *r*. Values of |*r*| ≥ 0.3 were considered moderate and |*r*| ≥ 0.5 strong effects. Results were visualized using boxplots. Variance homogeneity was assessed using Fligner-Killeen test with FDR correction, comparing patient-level summary statistics between regions.

Multiple linear regression models were used to assess associations between the quantitative parameters and age or months since completion of radiotherapy (RT). Regression coefficients, 95% confidence intervals, and *P-*value were estimated, and *P-*value were adjusted for multiple testing using an FDR correction.

## Results

**Figure 2.**
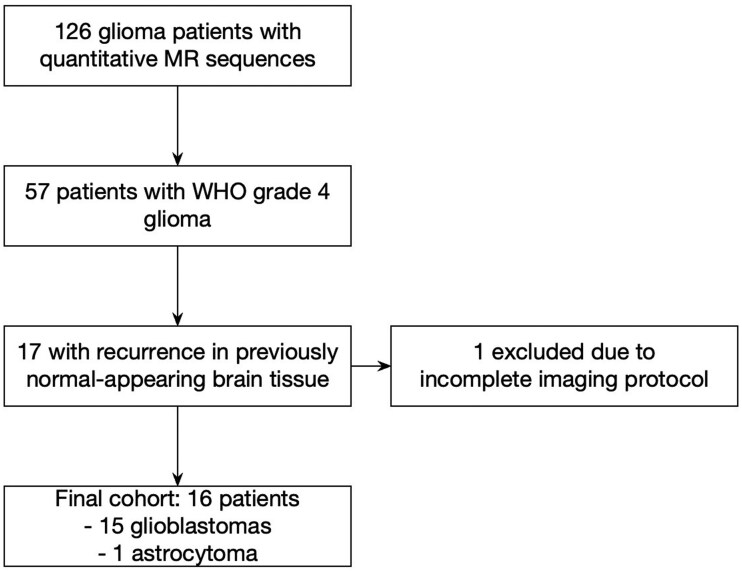
Flow diagram of the study cohort.

The tumors were classified according to the WHO Classification of Tumors of the Central Nervous System in effect at the time of diagnosis. The overall cohort comprised 126 patients with gliomas of WHO grades I-IV (2016 classification^24^) or 1-4 (2021 classification^25^) Among these patients, 57 had high-grade tumors corresponding to CNS WHO grade IV (2016) or CNS WHO grade 4 (2021) glioma. Based on a systematic review of the pathology reports, all tumors that had been graded as WHO grade IV fulfilled the criteria for CNS WHO grade 4 in the 2021 WHO classification. Within these 57 patients, 17 exhibited tumor recurrence in normal-appearing brain tissue, occurring within a maximum of 9 months following the quantitative MRI examination. One patient was excluded due to an incomplete scan protocol, resulting in a final study cohort of 16 patients [15 glioblastomas, 1 astrocytoma, CNS WHO grade 4; 4 women, 12 men; median age 58.6 (IQR 54.7-64.9) years]. About 15 out of the patients included in this study had received chemoradiation therapy prior to *t*_0_. About 14 were treated with a cumulative radiation dose of 60 Gy according to the Stupp protocol.^2^ One patient received a reduced dose of 40 Gy, and 1 patient had to undergo re-RT with 44 Gy. Recurrence was confirmed by histopathology in 8 cases, by PET imaging in 3 cases, and by radiological assessment according to RANO 2.0 criteria in 5 cases. The cohort selection process is summarized in a CONSORT-style flowchart in Figure 2.

The median interval between *t*_0_ and *t*_1_ was 3.0 (IQR 2.9-4.7) months, and the median time between completion of RT and recurrence was 6.75 (IQR 4.8-12.2) months. Patient characteristics are summarized in Supplementary Files (S2).

For the statistical analysis, only subregions with at least 100 voxels were included. Necrosis and CET were joined and labeled as “tumor,” as only a subset of patients had measurable tumor necrosis. Pooled median and IQR values of the different subregions for PD, R1, and R2* are summarized in Table 1.

**Table 1.** Medians and interquartile ranges (IQR) for the PD-, R1-, R2*-, and FW-values in the different subregions. p.u. = percent units

	PD [p.u.]	R1 [1/s]	R2* [1/s]	FW	FAt
	Median (IQR)	Median (IQR)	Median (IQR)	Median (IQR)	Median (IQR)
NAGM	76.0 (75.6-76.4)	0.62 (0.60-0.63)	19.8 (19.3-21.0)	0.22 (0.21-0.23)	0.25 (0.25-0.28)
NAGM/tumor	76.0 (73.8-77.1)	0.59 (0.54-0.62)	19.1 (15.7-22.1)	0.31 (0.23-0.39)	0.30 (0.26-0.37)
NAGM/FLAIR	74.8 (73.0-75.9)	0.60 (0.59-0.64)	19.6 (17.9-20.9)	0.25 (0.20-0.30)	0.28 (0.26-0.31)
NAWM	69.6 (69.5-69.7)	0.86 (0.81-0.87)	23.3 (22.5-24.3)	0.17 (0.15-0.18)	0.44 (0.43-0.45)
NAWM/tumor	69.3 (68.9-70.8)	0.81 (0.76-0.88)	23.1 (19.8-24.4)	0.25 (0.21-0.32)	0.49 (0.42-0.55)
NAWM/FLAIR	69.3 (68.9-70.1)	0.87 (0.83-0.89)	23.2 (21.7-24.8)	0.21 (0.16-0.24)	0.44 (0.42-0.48)

### Comparison of Quantitative MPM Values

**Figure 3.**
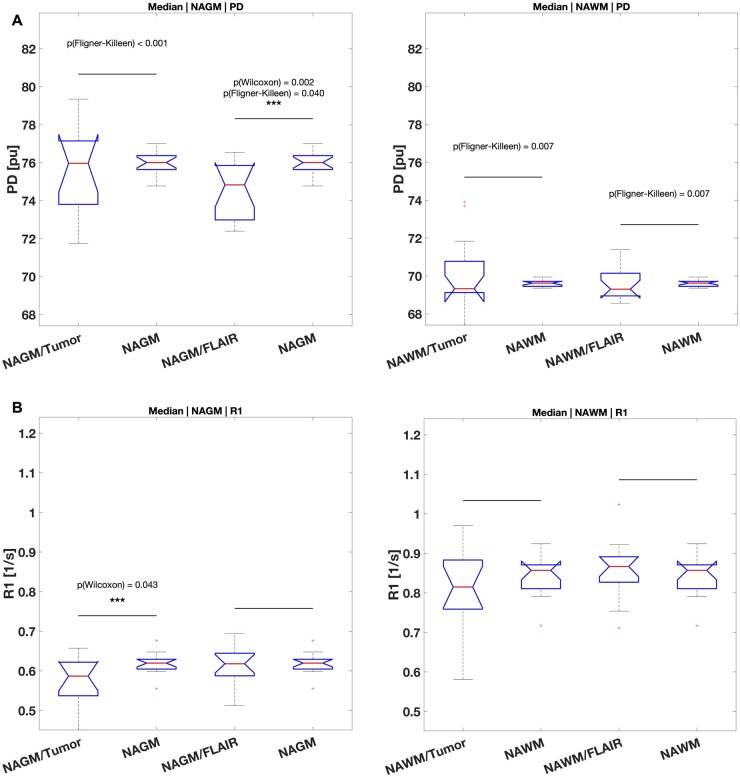
Boxplots visualizing the distribution of median PD (A) and R1 values (B) in the different subregions compared to NAGM (left column) and NAWM (right column). The horizontal lines within the boxes depict the median, the notched boxes indicate the IQR and the whiskers represent the range of non-outlier values. Horizontal brackets between paired box groups indicate statistical comparisons, annotated with *P-*values from Wilcoxon signed rank tests [*P*(Wilcoxon)] and Fligner-Killeen test for variance homogeneity [*P*(Fligner-Killeen)], both corrected for multiple comparisons using FDR correction. Statistically significant results of the Wilcoxon signed rank test (*P* < .05 after FDR correction) are annotated and visualized with ***.

Results of the PD- and R1 maps are plotted in Figure 3. PD values were significantly lower in NAGM/FLAIR compared to NAGM (*P* = .002). Variance analysis using Fligner-Killeen test with FDR correction revealed significant heterogeneity in median values between pathological and normal tissue regions. Specifically, NAGM/Tumor and NAGM/FLAIR exhibited significantly higher variance in PD median values compared to their corresponding normal tissue counterparts (*P* < .001 and *P* = .04, respectively). Similarly, median PD values in NAWM/Tumor and NAWM/FLAIR showed significantly higher variance compared to NAWM (both *P* = .007). Median R1 values in NAGM/Tumor were significantly lower than those in NAGM (*P* = .043).

**Figure 4.**
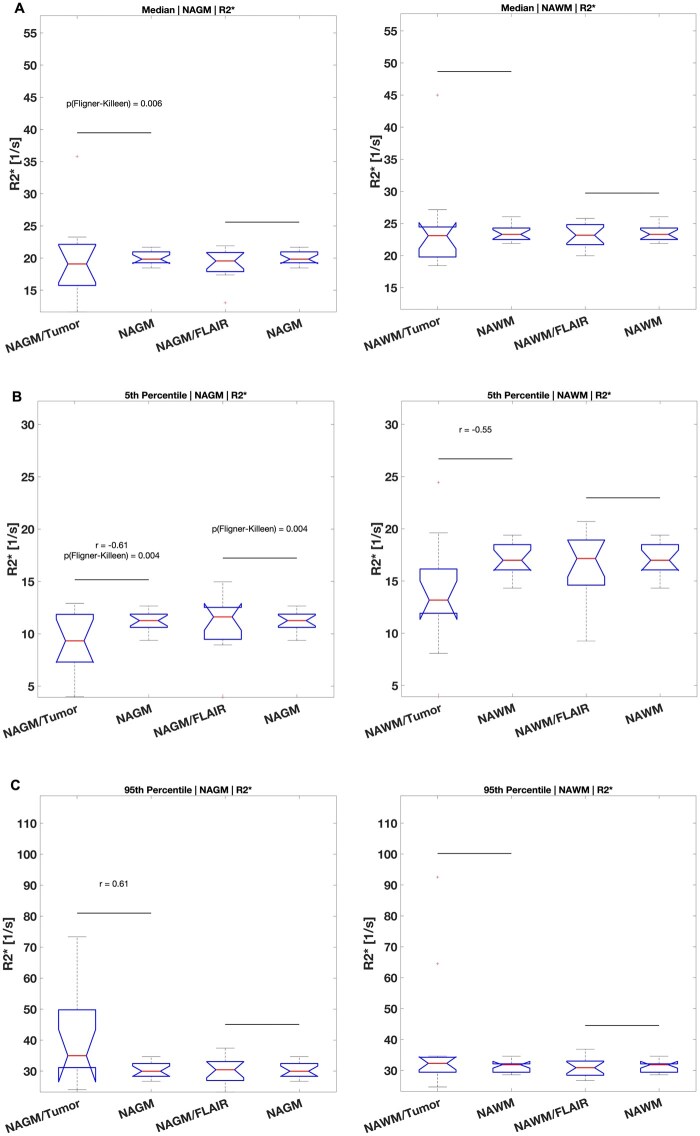
Boxplots visualizing median (A), the 5th percentile (B), and the 95th percentile (C) values of R2* (in 1/s) in the different subregions compared to NAGM (left column) and NAWM (right column). Wilcoxon signed-rank and Fligner-Killeen tests were performed. Correction for multiple testing was performed using FDR correction. For non-significant comparisons with substantial effect sizes, |*r*| > 0.5 are displayed to highlight clinically meaningful trends.

R2* analysis (Figure 4) did not reveal any significant differences in the Wilcoxon signed-rank tests. There were, however, strong trends toward lower P5 and higher P95 values in NAGM/Tumor compared to NAGM (*r* = −0.61, *r* = 0.61, respectively) as well as lower P5 values in NAWM/Tumor compared to NAWM (*r* = −0.55). Variance analysis showed significant heterogeneity in median and P5 values for NAGM/Tumor compared to NAGM (*P* = .006, *P* = .004, respectively).

### Comparison of Quantitative DTI-Derived Values

**Figure 5.**
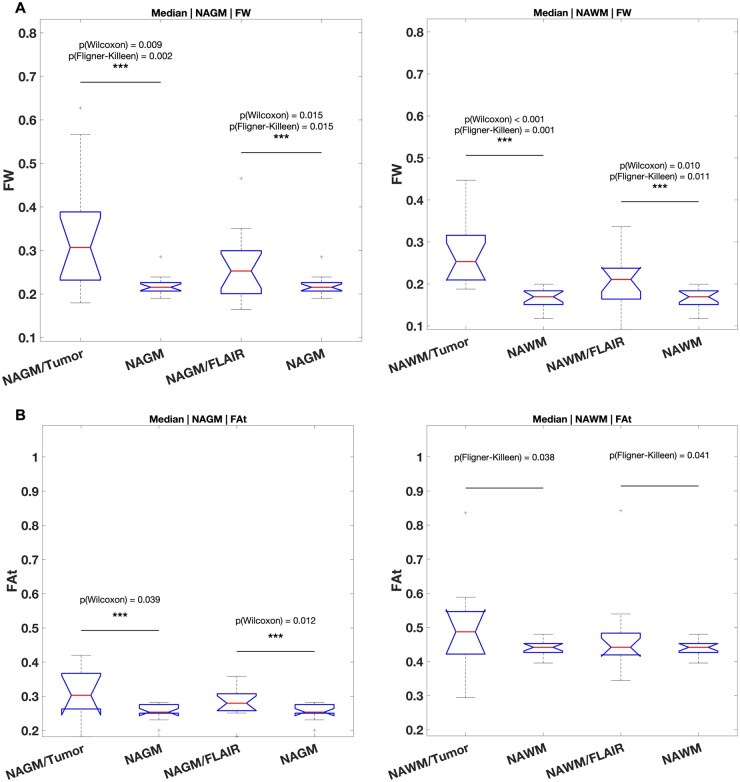
Boxplots visualizing the FW (A) and FAt (B) values in the different subregions compared to NAGM (left column) and NAWM (right column). Wilcoxon signed-rank and Fligner-Killeen tests were performed. Correction for multiple testing was performed using FDR correction. Statistically significant results of the Wilcoxon signed-rank test (*P* < .05 after FDR correction) are annotated and visualized with ***.

Figure 5 visualizes the results for the analysis of the FW and FAt maps. Median FW values were significantly higher in NAGM/Tumor and NAGM/FLAIR compared to NAGM (*P* = .009, *P* = .015, respectively) and in NAWM/Tumor and NAWM/FLAIR compared to NAWM (*P* < .001, *P* = .01, respectively). There was a significantly higher variance of FW values in all those regions (*P* = .002, *P* = .015, *P* = .001, *P* = .011, respectively). Median FAt was significantly higher in NAGM/Tumor and NAGM/FLAIR compared to NAGM (*P* = .039, *P* = .012, respectively). Variance was significantly higher in median values of NAWM/Tumor and NAWM/FLAIR compared to NAWM (*P* = .038, *P* = .041, respectively).

Multivariate regression analyses for PD, R1, R2*, FW and FAt did not show a statistically significant association with age or time since completion of RT after correction for multiple testing. In all four subregions (Tumor/NAGM, Tumor/NAWM, FLAIR/NAGM, FLAIR/NAWM), regression coefficients for age and months since RT were small, with adjusted *P-*value clearly above .05 (all FDR-corrected *P* ≥ .73 for age and ≥0.94 for months).

### Sensitivity Analysis

A sensitivity analysis was performed including only patients with histopathologically (*n* = 8) or PET-confirmed recurrence (*n* = 3), yielding a subgroup of 11 patients. Although the reduced sample size limited statistical power, the direction and magnitude of effects were largely preserved. Elevated median FW in NAWM/Tumor and lower median PD in NAGM/FLAIR remained statistically significant after FDR-correction. For median FW in NAWM/FLAIR and NAWM/Tumor as well as R1 in NAGM/Tumor, significance was not formally achieved, yet moderate to large effect sizes persisted which likely reflects the reduced sample size rather than a true absence of effect. Variance analysis showed preserved statistically significantly higher heterogeneity in median PD values in the NAWM/Tumor region. Median R2* values in NAGM/Tumor also exhibited higher variance than NAGM, with a borderline effect that narrowly missed significance after FDR correction (*P* = .03, *P*_FDR_ = .05).

## Discussion

In this study, we investigated whether quantitative PD, R1, and R2*, as well as DTI-derived FW and FAt, can detect measurable changes in radiologically normal-appearing brain tissue that subsequently progresses to recurrence in high-grade glioma. The most compelling finding was a consistent elevation of FW across all future tumor subregions, accompanied by lower median PD values in NAGM—a pattern compatible with an early compartmental redistribution of water from bound tissue pools into the freely diffusing extracellular space. Additionally, R1 was significantly reduced in NAGM destined to become contrast-enhancing tumor, suggesting early occult tumor infiltration. R2* showed strong trends toward both elevated P95 and reduced P5 values, consistent with spatially heterogeneous microenvironmental changes encompassing focal hypoxia and increased free water. Increased variance across multiple parameters further underscores the heterogeneous nature of early infiltration.

### Quantitative Values in the Literature

In our cohort, PD and R1 values in NAWM and NAGM were in a similar range to published reference data for healthy volunteers, whereas R2* values were slightly higher. Several factors limit direct comparison between reference values and our patient cohort. Weiskopf et al enrolled only 5 young, healthy volunteers (mean age 24 ± 1.6 years),^26^ while Neeb et al analyzed 16 healthy volunteers between the ages of 20 and 40.^27^ In contrast, our patients represent an older demographic with potential age-dependent alterations. More substantially, normal-appearing brain tissue in glioblastoma patients is not simply “normal” but may harbor therapy-related changes and tumor-related pathology such as subclinical infiltration that is not present in healthy volunteers. Consequently, our observations in regions of subsequent tumor progression should primarily be interpreted as within-study comparative findings rather than as direct deviations from published norms.

Quantitative MR parameters are influenced by RT and physiological aging in addition to tumor-related changes: Ljusberg et al demonstrated that both PD- and R2-values significantly changed after RT and exhibited dosage-dependent effects. Specifically, they observed higher values for both parameters in the first follow-up scan. While these changes gradually returned to baseline in subsequent scans for radiation doses <30 Gy, PD values continued to increase over time for doses ≥30 Gy, indicating progressive tissue alteration in high-dose areas.^28^ Callaghan et al analyzed the effect of age on quantitative parameters in healthy volunteers. They reported negative correlations between PD and age in the basal ganglia and positive correlations in superior white matter regions. R1 decreased in specific structures, such as the optic radiation, with a range of 0.0007 to 0.0016 1/s per year. R2* increased by 0.03 to 0.22 1/s per year and showed location-specific differences with structures like the basal ganglia exhibiting higher values.^29^

Blystad et al report values in NAWM on the contralateral side of the tumor. In their study, they did not analyze R2* but R2 values.^12^ While their measured PD values are comparable to ours, there is a large discrepancy in R1 values. This likely stems from methodological differences: Blystad et al placed a manually drawn region of interest in visually homogeneous, contralateral NAWM. Our whole-brain NAWM segmentation includes potentially mildly altered but visually normal white matter, which might account for the lower median R1 values.

### PD and FW May Reveal Potential Shifts in Water Compartments

PD serves as a quantitative surrogate for tissue water content. In our cohort, PD values in NAGM and NAWM agreed well with published data for grade 3 and 4 glioma patients.^12^ Prior work by Reuter et al reported elevated PD values in postoperative T2/FLAIR-hyperintense regions that later developed tumor recurrence compared to FLAIR abnormalities without subsequent progression.^30^ In the present study, such visibly altered regions were systematically excluded, and the analysis was restricted to normal-appearing brain tissue.

At first glance, the combination of lower PD and higher FW in NAGM that becomes FLAIR-hyperintense in the follow-up examination appears contradictory. However, this apparent discrepancy is resolved when considering the fundamentally different water compartments that each metric captures. PD reflects the total MRI-visible water content of a voxel, encompassing intracellular, extracellular, and myelin-bound water pools.^31^ In contrast, the DTI-derived FW fraction quantifies exclusively the volume fraction of isotropically and freely diffusing extracellular water within a bi-tensor model fitted to single-shell DTI data. Consequently, a redistribution of water between compartments might account for a change in opposite directions.^32^^,^^33^ Our results differ conceptually and technically from intravoxel incoherent motion (IVIM) metrics, which require multi-b-value acquisitions and primarily capture microvascular perfusion-related signal components.^34^^,^^35^

We therefore interpret our findings as compatible with an early, regionally heterogeneous redistribution of water from bound and restricted compartments (myelin, intracellular space) to the extracellular space. Mechanistically, this could involve cell shrinkage as invading glioma cells undergo a hydrodynamic volume reduction of approximately 30%-35% as they navigate the narrow extracellular spaces of the brain parenchyma.^33^ The process of tumor cell shrinkage involves the efflux of K^+^ and Cl^-^ ions through chloride- and calcium-activated potassium channels. Water molecules consequently follow passively through aquaporin channels.^36^ The released intracellular water enters the extracellular compartment as freely diffusing water, directly increasing the FW fraction. Preclinical evidence supports this mechanism: invasive glioma cells show enlarged extracellular volume fractions correlating with aquaporin overexpression,^37^ and glioma-driven upregulation of hyaluronic acid further expands the extracellular space and increases its water-binding capacity.^38^

Second, tumor infiltration disrupts the microstructural integrity of normal brain tissue, including progressive demyelination.^39^ Myelin water, which constitutes approximately 11% of total white matter water and 3% in gray matter,^31^ is therefore lost during this process, reducing the overall tissue water content and consequently PD.

Taken together, the 2 mechanisms would decrease total voxel water content (lower PD), while increasing the relative proportion of freely diffusing extracellular water (higher FW), particularly in gray matter that later develops vasogenic edema. The increased variance in both PD and FW within future tumor regions further supports a heterogeneous mixture of microenvironments rather than a uniform shift.

### R1 and R2* as Early Markers of Tumor-Related Tissue Alterations

Increased T1 relaxation times (eg lower R1 values) were observed in therapy-naive high-grade glioma^40^ and recurrent glioblastoma.^41^ Consistent with this pattern, our analysis revealed significantly lower median R1 values in NAGM/Tumor compared to NAGM. We interpret these subtle R1 reductions as potential indicators of early occult tumor cell infiltration into normal-appearing brain regions.

Though not significant, the R2* analysis revealed a strong trend toward higher P95 values in NAGM/Tumor compared to NAGM, suggesting the presence of focal high-susceptibility outliers. High R2* values are typically interpreted as a surrogate for increased deoxyhemoglobin concentration and reduced tissue oxygenation. In the context of brain tumors, this might stem from regional hypoxia driven by rapid cellular proliferation in high-grade gliomas.^42^ Increased microvessel density, a hallmark of glioblastoma angiogenesis, leads to a higher absolute amount of deoxyhemoglobin per voxel. This aligns with findings by Maurer et al., who observed lower T2* (ie higher R2*) values in histological areas of high vessel density.^40^ Furthermore, micro-hemorrhages frequently associated with aberrant tumor neovascularization introduce paramagnetic hemosiderin, potentially further elevating R2* values independent of the current metabolic state.^43^

R2* mapping has been shown to differentiate between glioma grades, with high-grade gliomas exhibiting significantly higher R2* values compared to low-grade tumors.^44^ Regarding recurrence specifically, Reuter et al reported increased R2* values in CET arising within postoperative T2/FLAIR-hyperintense areas compared to those emerging in radiologically normal-appearing tissue. Stadlbauer et al detected increased hypoxia as a potential early biomarker of glioblastoma recurrence.^45^

Conversely, the observed trend toward lower P5 values of R2* in NAGM/Tumor vs NAGM and NAWM/Tumor vs NAWM suggests the coexistence of opposing microenvironmental states. Areas with increased free water accumulation, as evidenced by the elevated FW fractions reported above, would be expected to dilute susceptibility effects and lower local R2* values.

The significant variance detected in R2*values further underscores the coexistence of these contrasting tissue microenvironments within tumor-infiltrated regions, consistent with the known spatial heterogeneity of glioblastoma microvasculature and oxygenation status.^46^

### FAt Might Not Be Suitable to Detect Subtle Changes in Normal-Appearing Brain Tissue

FA is widely interpreted as a marker of microstructural organization, reflecting the coherence and directional alignment of tissue structures that constrain water diffusion. In highly organized white matter, high FA is typically associated with greater fiber coherence and reduced orientation dispersion, whereas decreased FA reflects either axonal loss, demyelination, or increased architectural complexity such as crossing fibers.^47^ FAt measurements become more accurate, as the confounding isotropic contribution of extracellular fluid is removed^20^ and is able to better predict glioblastoma recurrence within preexisting FLAIR-hyperintense areas than FA.^48^

In the present cohort, FAt values were significantly higher in NAGM that later progressed to tumor or FLAIR-hyperintense areas, which is counterintuitive given that early infiltration would be expected to reduce anisotropy. In NAWM, many recurrences involved the corpus callosum, where highly coherent fiber architecture likely drives FAt upward irrespective of subtle early damage. In NAGM, partial-volume effects from adjacent white matter tracts at the cortical—subcortical boundary may have contributed to elevated FAt. Together with the increased variance of FAt in NAWM that later progressed, these findings suggest that FAt is dominated by baseline tissue organization rather than subtle infiltrative change, limiting its sensitivity as an early biomarker in radiologically normal-appearing tissue. FAt may therefore be more suitable for differentiating edema from dense tumor in already abnormal regions. We therefore do not recommend FAt as an early recurrence biomarker in radiologically normal-appearing tissue.

RT received prior to *t*_0_ may contribute to some of the observed differences in variance. However, in our study, multivariate analyses showed that, within the limited sample of 16 patients, regional quantitative values were not detectably influenced by age or time since RT. This absence of a significant association with known clinical covariates suggests that the observed differences between future tumor and reference regions may reflect early, radiologically occult tumor progression rather than confounding effects of patient age or RT.

Importantly, our results were mostly preserved in a sensitivity analysis restricted to patients with histological or PET-confirmed recurrence, supporting the robustness of our findings despite the small sample size. Of note, the 5 patients with radiologically confirmed recurrence had all been discussed in a multidisciplinary tumor board and demonstrated imaging features characteristic of progression, including restricted diffusion and hyperperfusion of at least part of the contrast-enhancing region. The exclusion of the 5 patients therefore represents a conservative scenario. Nevertheless, a contribution of post-treatment effects in addition to true tumor recurrence cannot be definitively excluded.

### Clinical Feasibility and Translational Implications

The clinical utility of quantitative MRI is critically dependent on its feasibility within routine workflows. Recent advances have substantially reduced acquisition time, and the MPM protocol used in the present study can be easily integrated into standard follow-up MRI sessions without significant additional patient burden.

Early detection of subclinical tumor infiltration could serve concrete purposes. First, it may refine risk stratification during surveillance: individuals showing MPM alterations in normal-appearing tissue could be flagged for shortened follow-up intervals, analogous to how amino acid PET findings currently guide surveillance in unclear cases.^49^ Second, quantitative imaging biomarkers could inform RT planning. There is a growing body of work on margin strategies and adaptive RT planning in high-grade gliomas. However, they rely on conventional MRI.^50^ Adding MPM could further refine target volume planning by identifying regions of radiologically normal-appearing tissue at higher risk for future recurrence.

### Limitations

While this study provides valuable insights into early subtle changes in recurrent high-grade glioma, several limitations should be acknowledged. First, the relatively small cohort size of 16 patients may limit the generalizability of our findings and the statistical power to detect associations with clinical outcomes. Second, 1 patient was diagnosed with high-grade astrocytoma rather than glioblastoma, and some cases were originally classified under the 2016 WHO system; however, all tumors would be graded WHO 4 according to the 2021 classification, ensuring consistent grading across the cohort. Third, combining necrosis and CET in the analysis might introduce some form of bias, as necrosis can also be induced by RT. Yet, given that necrosis typically coexists with recurrent tumor in high-grade gliomas, we consider this composite definition to reflect routine clinical practice and its potential impact on our results to be limited. Fourth, the absence of histopathological validation for the observed changes in normal-appearing tissue precludes direct confirmation of the underlying molecular and cellular correlates. Lastly, the subtle changes identified in this study may have a limited direct clinical impact on patient management or therapeutic decision-making at present. However, they may prove valuable as biomarkers for identifying high-risk patients and refining surveillance strategies. Despite these limitations, our findings contribute to the growing body of evidence aimed at improving early detection of high-grade glioma recurrence and optimizing personalized treatment approaches.

## Conclusion

Subtle changes in normal-appearing brain tissue that subsequently become a tumor region occur several months before being visible in standard MRI sequences. Quantitative MRI maps and DTI-derived metrics could help identify subclinical progression, enabling improved prognostication and earlier therapeutic decision-making.

## Supplementary Material

vdag173_Supplementary_Data

## Data Availability

The data underlying this article will be shared on reasonable request to the corresponding author.
