# Supplementary material for: Detection of early alterations in radiologically normal-appearing brain regions before recurrence of high-grade glioma using multiparameter mapping and diffusion tensor imaging
Source: Neurooncol Adv. 2026 Jul 2;8(1):vdag173. doi: 10.1093/noajnl/vdag173 (PMC13385338; doi:10.1093/noajnl/vdag173)
Supplement: vdag173_Supplementary_Data [file vdag173_supplementary_data.docx]

**Supplements**

S1: Imaging protocol

All MRI scans were performed on a Philips Ingenia Elition 3.0 Tesla (Philips Medical Systems, Hamburg, Germany). Standard diagnostic MRI consisted of a T1‐weighted (T1w) magnetization‐prepared rapid gradient‐echo sequence before and after application of gadolinium-based contrast agent (voxel size = 1 × 1 × 1 mm^3^, echo time (TE)=4 ms, repetition time  (TR)=9 ms, inversion time (TI)=761 ms*,*flip angle=8°, 267 slices, each), a native T2-weighted (T2w) (voxel size 1 × 1 × 1 mm^3^, TE=280 ms, TR=3000 ms, flip angle=90°, 260 slices), a FLAIR sequence (voxel size 1×1×1 mm^3^, TE=325 ms, TR=4800 ms, TI=1650 ms, flip angle=90°, 281 slices) and a DSC perfusion with a gradient‐echo echo planar imaging readout, voxel size of 1.75×1.75 × 4 mm^3^ (TE=40 ms, TR=1602 ms, flip angle=75°, 25 slices). Diffusion tensor imaging (DTI) was acquired using a spin-echo echo-planar imaging sequence (voxel size 1.75x1.75x2 mm^3^, TE=77 ms, TR=3800 ms, flip angle 90°, b=1000 s/mm^2^, 2376 slices). The quantitative acquisitions included a previously established accelerated multiparameter mapping (MPM) protocol.^7^

- B1 mapping: TR=30/150 ms, TE=2.34ms, a=60°, 70 slices, voxel size 3.5x3.5x3.5mm^3^, Compressed SENSE (CS) factor=6.
- T1-weighted multi echo Turbo Field Echo (TFE): TR=18ms, 6 echoes, TE/DTE=2.4ms, a=25°, CS factor=6.
- PD-weighted multi echo TFE: TR=18ms, 6 echoes, TE/DTE=2.4ms, a=25°, CS factor=6.

The B1 map was used to assess and correct radiofrequency field inhomogeneities. The T1- and PD-weighted acquisitions were obtained with identical geometry (176 slices, voxel size 1x1x1 mm^3^) to enable voxel-wise quantitative T1 mapping.

The follow-up scans (only conventional MRI) were performed on one of the following scanner types: Philips Ingenia 3.0T, Philips Achieva 3.0T, Philips Ingenia Elition 3.0T (Philips Medical Systems, Hamburg, Germany), or Siemens Magnetom Avanto 1.5T (Siemens Healthineers, Erlangen, Germany). The minimally necessary MRI protocol consisted of a 3D FLAIR and a contrast-enhanced 3D T1 MRI.

| Variable | Overall (n=16) | Glioblastoma (n=15) | Astrocytoma (n=1) |
| --- | --- | --- | --- |
| Age, median (IQR), years | 58.6 (54.7-64.9) | 59.4 (54.8-64.9) | 47.6 |
| Female, n (%) | 4 (25%) | 4 (26.7%) | 0 (0%) |
| IDH status  Wild type n (%) | 15 (93.8%) | 15 (100%) | 0 (0%) |
| Mutation n (%) | 1 (6.2%) | 0 (0%) | 1 (100%) |
| MGMT  methylated, n (%) | 6 (37.5%) | 5 (33.3%) | 1 (100%) |
| not methylated, n (%) | 10 (62.5%) | 10 (66.7%) | 0 (0%) |
| Received chemoradiation therapy prior to t_0_ | 15 (93.8%) | 14 (93.3%) | 1 (100%) |
| Cumulative RT dosage in Gy before t_0_,  median (IQR) | 60 (60-60) | 60 (60-60) | 40 |
| Months between prior RT and t_0_, median (IQR) | 6.75 (4.8-12.2) | 6.9 (5-12.5) | 4.3 |
| Months between t_0_ and t_1_, median (IQR) | 3 (2.9-4.7) | 3 (2.8-4.7) | 3.4 |
| Confirmation of recurrence |  |  |  |
| Histopathology, n (%) | 8 (50%) | 8 (53.3%) | 0 (0%) |
| PET imaging, n (%) | 3 (18.8%) | 3 (20%) | 0 (0%) |
| RANO 2.0, n (%) | 5 (31.2%) | 4 (26.7%) | 1 (100%) |

S2: Patient cohort characteristics

**Figure legends**

Figure 1**:** 59 year old male patient with glioblastoma WHO CNS grade 4: Routine scans for response assessment (t_0_) with no signs of tumor recurrence in axial FLAIRw sequence (A) and T1w post contrast (T1c, B). Stable disease according to RANO 2.0. (D+E) The same patient 3 months later (t_1_). Extensive tumor recurrence with new FLAIR-hyperintensities, CET and necrosis in the right frontal lobe (see red arrows). Progressive disease according to RANO 2.0. (C) Fused segmentation of subregions shows normal-appearing tissue at t_0_ that will progress to tumor regions at (t_1_). Note that pre-existing FLAIR-hyperintensities in the right frontal lobe and adjacent to the right lateral ventricle were manually excluded. (F) shows the different subregions. The violet label, for instance, depicts NAGM in the routine scan, which became CET in the follow-up scan. Quantitative maps at t_0_:(G) PD map, (H) R1 map, (I) R2* map. Note the susceptibility-artefacts close to the frontal sinuses in the R2* map (yellow arrow). DTI-derived metrics at t_0_: FAt map (J), FW map (K).

Figure 2: Flow diagram of the study cohort.

Figure 3: Boxplots visualizing the distribution of median PD (A) and R1 values (B) in the different subregions compared to NAGM (left column) and NAWM (right column). The red lines depict the median, the notched boxes indicate the IQR and the whiskers represent the range of non-outlier values. Horizontal brackets between paired box groups indicate statistical comparisons, annotated with p-values from Wilcoxon signed rank tests [p(Wilcoxon)] and Fligner-Killeen test for variance homogeneity [p(Fligner-Killeen)], both corrected for multiple comparisons using FDR correction. Statistically significant results of the Wilcoxon signed rank test (p < 0.05 after FDR correction) are annotated and visualized with ***.

Figure 4: Boxplots visualizing median (A), the 5^th^ percentile (B) and the 95^th^ percentile (C) values of R2* (in 1/s) in the different subregions compared to NAGM (left column) and NAWM (right column). Wilcoxon signed-rank and Fligner-Killeen tests were performed. Correction for multiple testing was performed using FDR correction. For non-significant comparisons with substantial effect sizes, |r| > 0.5 are displayed to highlight clinically meaningful trends.

Figure 5: Boxplots visualizing the FW (A) and FAt (B) values in the different subregions compared to NAGM (left column) and NAWM (right column). Wilcoxon signed-rank and Fligner-Killeen tests were performed. Correction for multiple testing was performed using FDR correction. Statistically significant results of the Wilcoxon signed rank test (p < 0.05 after FDR correction) are annotated and visualized with ***.
